# Warfarin-associated intracranial haemorrhage in pregnant woman with double mechanical valve replacement: a case presentation

**DOI:** 10.1186/s12872-020-01547-5

**Published:** 2020-06-11

**Authors:** Mustafa Oguz, Ahmet Ayaz, Mehmet Emin Adin

**Affiliations:** 1 Department of Cardiology, University of Health Sciences, Van Research and Training Hospital, Cardiology Clinic-Süphan, Neighborhood Airport, Intersection 1. Kilometer, Edremit / Van, Turkey; 2 Department of Radiology, Koc University, Istanbul, Turkey

**Keywords:** Major bleeding, Intracranial haemorrhage, Pregnant woman, Prosthetic valve replacement, Case presentation

## Abstract

**Background:**

Management of warfarin-associated major haemorrhage in prosthetic valve diseases is difficult as there is a fine line between haemorrhage and thrombosis. An individual’s propensity towards thrombosis, such as pregnancy, makes this situation even more complicated. Cases like these are very rare in the literature**.**

**Case presentation:**

A 26 weeks pregnant, gravida two, para one, 35-year-old patient with prosthetic aortic and mitral valves presented to an external emergency clinic with clouding of consciousness. Her international normalised ratio(INR) was 8.9 at presentation. Brain MRI revealed a left subdural haematoma with no significant mass effect. Warfarin treatment was discontinued. On the second day of follow-up, she was referred to our centre for further evaluation of her clinical deterioration. She was haemodynamically stable on admission to the intensive care unit and followed up with a stable condition until the fourth day when she developed right eye drop and subsequent loss of consciousness. Her haematoma was surgically evacuated, and her condition improved. Eventually, she and a healthy newborn were discharged.

**Conclusion:**

Intracranial haemorrhage during pregnancy is a relatively rare complication that requires a multidisciplinary management plan. Although the thrombogenic risk is high, it is vital to complete a reversal of warfarin anticoagulation in pregnant women with major bleeding.

## Background

Management of intracranial haemorrhage (ICH) in pregnant women on anticoagulation is not well described in clinical guidelines. In all prosthetic valve patients, 50% of the haemorrhages are major, and they have a mortality rate of 9.5–13.4% [1, 2]. However, there is data available for major bleeding in pregnant patients, and the ICH mortality rate is likely even higher in high-risk patients (pregnant and double-valve replacement patients). In pregnant patients, the haemorrhage antidote type, dose, and timing for stopping; correcting the anticoagulant effect; and the ideal timing for restarting warfarin are all still controversial. This ambiguity complicates everyday clinical practice. Here, we report a case of ICH in a pregnant woman with prosthetic aortic and mitral valves.

## Case presentation

**Fig. 1:**
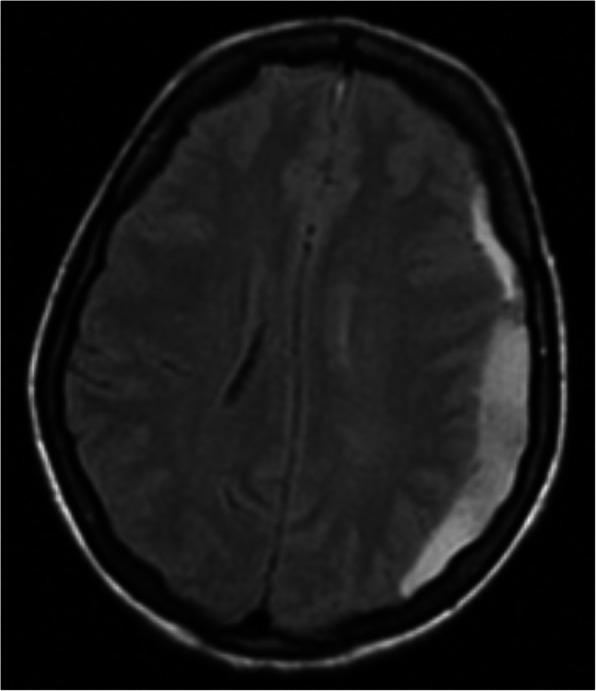
Axial T2-weighted-Fluid-Attenuated Inversion Recovery (FLAIR) image shows left subdural hematoma causing slight right midline shift

**Fig. 2:**
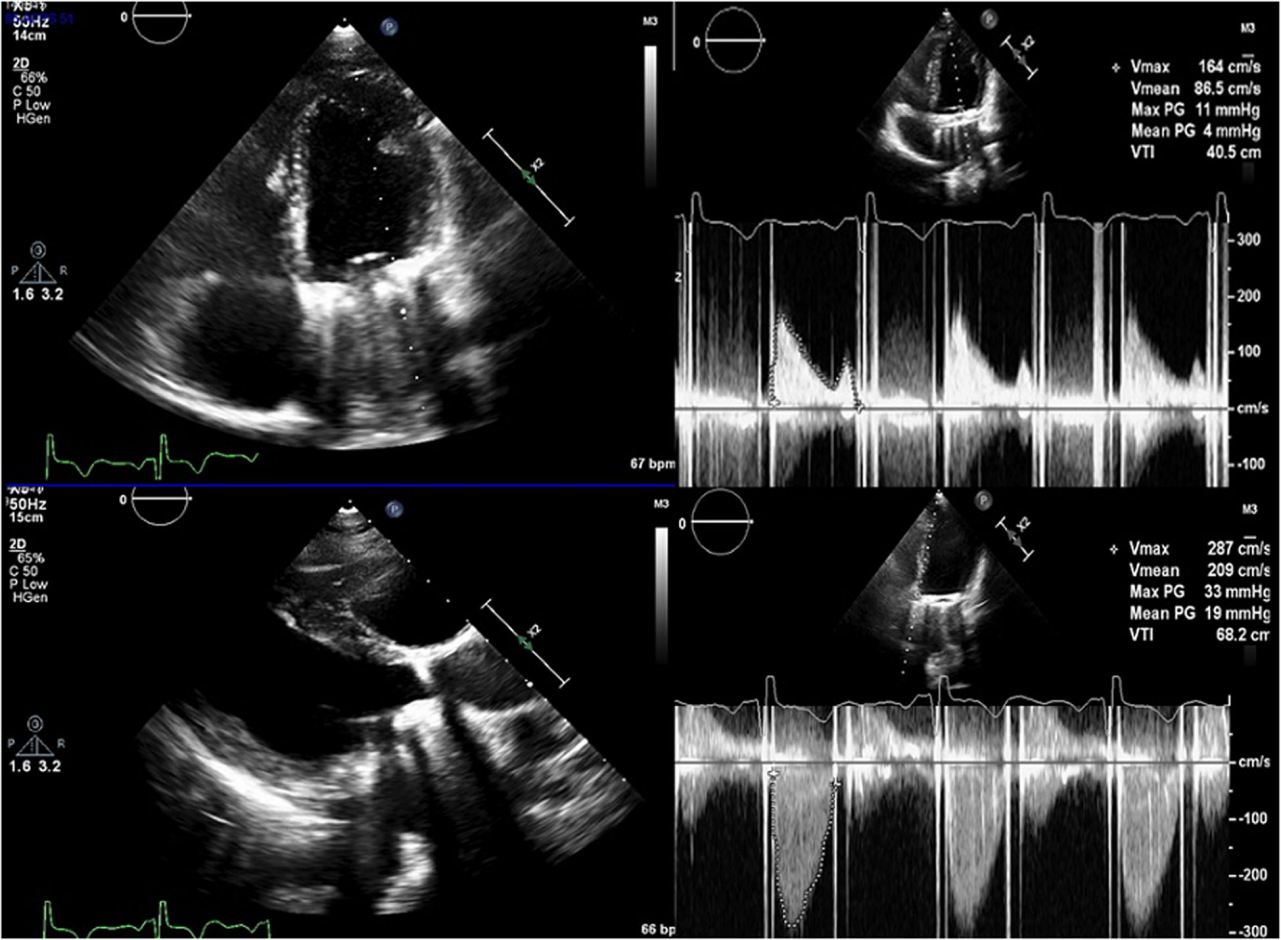
Parasternal Long Axis Echocardiographic view shows Mechanical Aortic and Mitral Valve with no identifiable thrombus formation. Parameters of Mechanical Aortic and Mitral Valve as seen apical 4 and 5 chamber echocardiographic view

**Fig. 3:**
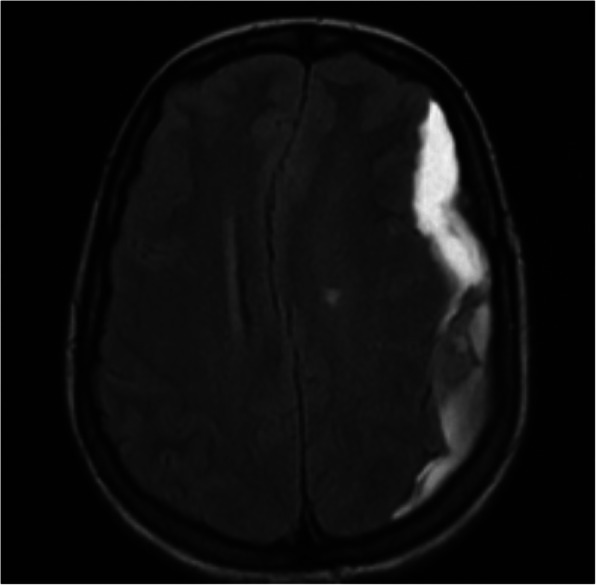
Axial T2-weighted-Fluid-Attenuated Inversion Recovery (FLAIR) preoperation image shows expansion of left subdural hematoma causing severe right midline shift

**Fig. 4:**
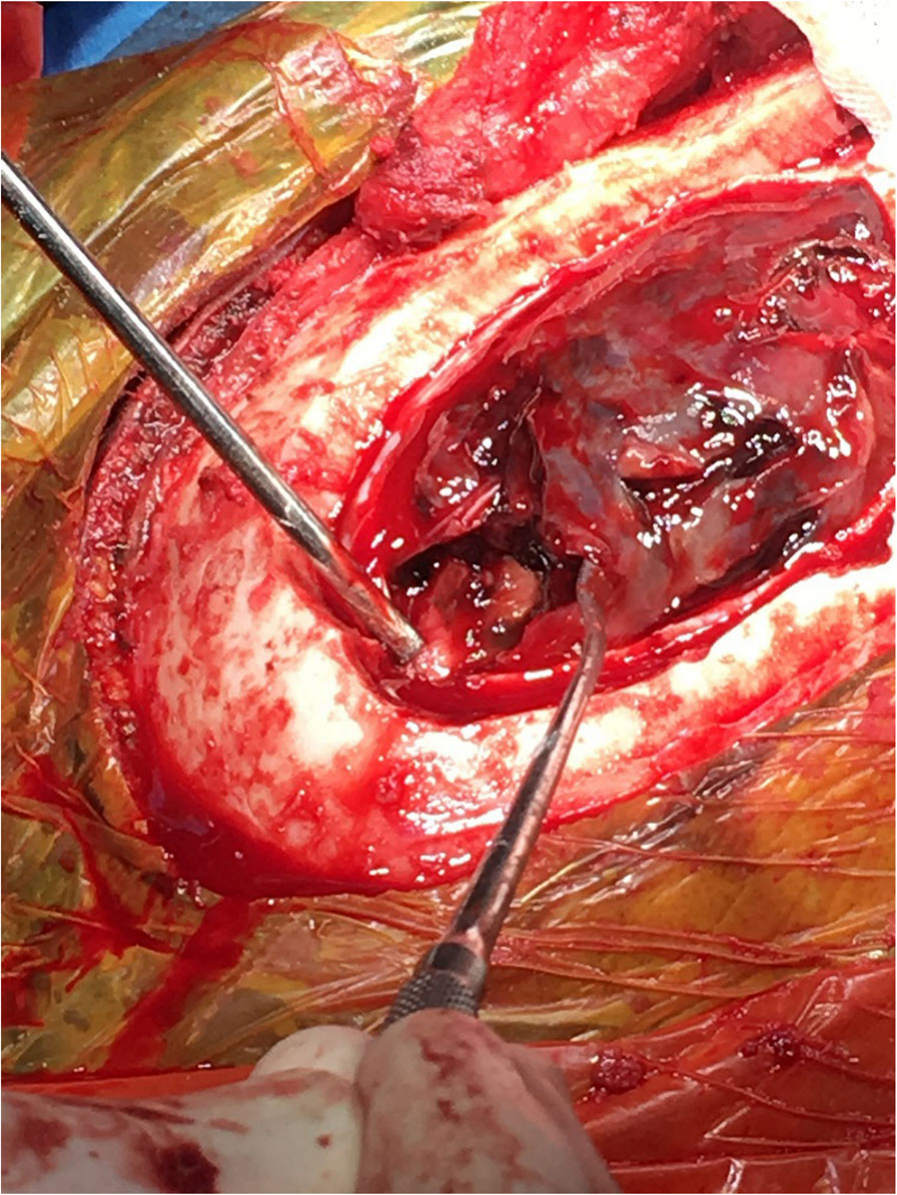
Craniotomy of the Patient Image shows organized subdural hematoma

A 26 weeks pregnant, gravida two, para one, 35-year-old patient, operated on due to rheumatic heart valve disease 12 years ago with bileaflet prosthetic aortic and mitral valves, presented to an external emergency clinic with clouding of consciousness and ongoing complaints of nausea and vomiting for 2 weeks (Glasgow Coma Scale [GCS] 13). A subdural haematoma was detected on brain MRI (Fig. 1). On admission, the patient’s international normalised ratio (INR) was 8.9, and she had been receiving 5 mg warfarin daily for the last 5 weeks. With a healthy pregnancy, she was coumadinized 12 weeks after enoxaparin treatment in the first trimester, and her INR was being followed up once every 2 weeks, except for skipping the most recent INR testing due to social problems. The time in therapeutic range was 66.4 between 12 and 26 weeks of pregnancy with a 2.5–5.0 mg warfarin dose. After warfarin treatment was discontinued, 1/2 ampule IV (5 mg) vitamin K was applied, and the controlled INR was 7.2. She was then admitted to an external centre intensive care unit. Her physical exam revealed mild bibasilar rales on a lung examination. Surface ECG revealed 124/mn sinus tachycardia. Her clinical condition deteriorated haemodynamically with tachycardia and hypotension on the second day of follow-up, so she was referred to our centre for further evaluation. A consulting cardiologist, neurosurgeon, neurologist, and neonatologist team evaluated the patient on admission. At the initial evaluation, the patient had clear consciousness without neurological deficits (GCS of 15), and she was haemodynamically stable. The admission INR was 6.7. After a joint discussion with the consulting specialists, both the mother and foetus were accepted as vitally stable. Her mechanical valves were functional, with no identifiable thrombus formation on echocardiographic evaluation (Figs. 2 and 3). We considered the reversal of warfarin anticoagulation with vitamin K or fresh frozen plasma (FFP). However, although we were concerned about the potential risk of rebound hypercoagulability leading to valvular thrombosis, heart failure, thromboembolism, or even maternal mortality [3, 4], the cardiology and neurosurgery team decided to wait and watch because the patient was haemodynamically and neurologically stable. A spontaneous fall of INR was expected with daily INR work-up. Unfortunately, her INR was 1.67 on day four of follow-up, and she suddenly developed right eye drop followed by loss of consciousness. She immediately underwent brain MRI that demonstrated an interval expansion of the subdural hematoma compared with the previous brain MRI (Fig. 3). Immediate surgical intervention was warranted due to a midline shift caused by a mass effect from the subdural collection. The subdural haematoma was uneventfully evacuated through a right parietal craniotomy (Fig. 4), and her consciousness returned soon after the operation was completed. On the fourth day after the operation, unfractionated heparin (UFH) was started with an infusion rate of 1000 U/hr. after a 5000 U intravenous bolus. The aPTT value was adjusted to between 50 and 70 s. The patient was then followed up with UFH infusion for 3 days. No evidence of thrombosis was found in the prosthetic valves on daily echocardiographic evaluation. On the seventh day after surgery, 2 × 0.6 cc subcutaneous enoxaparin was given, and the level of anti-factor Xa was tested. Because the patient’s condition had improved, she was discharged from the hospital after her anti-factor Xa level reached 1.1 IU/mL, and weekly anti-Xa level monitoring (therapeutic range 0.8–1.2 IU/mL) was recommended, with a prescription of twice daily 0.6 cc subcutaneous enoxaparin. Her baby was delivered at the 37th week of pregnancy via an uncomplicated Cesarean delivery under UFH treatment. The newborn was healthy and appropriate for gestational age. The patient was again coumadinized and discharged with recommendations (goal INR of 3–3.5).

## Discussion and conclusion

Pregnancy in women with mechanical valves is associated with a very high risk of complications, such as valve thrombosis, thromboembolism, and bleeding (WHO risk class III) [5]. However, there is no consensus in the current guidelines on the treatment protocol of warfarin-induced major haemorrhage in pregnant patients with mechanical prosthetic valves, and studies mainly consist of case presentations [3]. The estimated risk of thromboembolic complications in patients with prosthetic heart valves during pregnancy ranges from 7 to 23%, and the valve thrombosis mortality rate can be up to 40% [6]. However, trends in the Ropac registry show that for valvular heart disease, there has been a decrease in maternal mortality: 7% of patients with a mechanical valve suffered from valve thrombosis, of which 18% died [7].

Reversing anticoagulation during intracranial bleeding is challenging in the presence of a prosthetic heart valve because the risk of thrombotic complications increases during pregnancy. Treatment options, such as vitamin K, FFP, prothrombin complex concentration, and recombinant active factor VIIa are used to stop bleeding, but their use is limited because of a resulting mortality rate of up to 40% due to rebound hypercoagulability, which increases the risk of mechanical valves thrombosis (MVT) in pregnancy [8]. However, for major bleeding, haemorrhagic shock, and urgent emergency surgery situations, the reduction of INR is advised by means of vitamin K or FFP [8]. Our case was within a high-risk group in terms of high thrombogenicity due to double valve replacement and pregnancy. At the time of admission, our case was expectantly managed for a spontaneous decrease in INR due to the high thrombogenic risk and the patient’s stability, even though the INR was as high as 6.7. However, after the INR fell to 1.67, there was a sudden deterioration in her clinical condition, attributed to the slow expansion of an underlying hematoma that necessitated urgent intervention.

It is not clear which anticoagulant should be initiated and when to start it after a major surgical operation for a pregnant patient with mechanical valve disease. Phan et al.’s [9] study of mechanical valve patients found that ICH patients had a 30-day thromboembolic risk of 3% on day 10 of anticoagulant withdrawal. In another study, no MVT was seen in patients without anticoagulation from 2 days to 3 months (a mean of 8 days) [10]. The AHA guideline [11] recommends that warfarin be restarted after 7–10 days in ICU, while the European Stroke Initiative committee recommends it is restarted after 10–14 days [12]. All anticoagulants can be eliminated during these periods, so the use of Low-molecular-weight heparine or UFH as a bridge therapy is also recommended after bleeding control is achieved [13]. Patients should not be rapidly re-coumadinized or started on anticoagulants in the early stages of haemorrhage because of the low risk of MVT during short-term warfarin withdrawal relative to the risk of re-haemorrhage, which has a higher risk scale and is more frequently observed. However, due to the high thrombogenic susceptibility of our patient, UFH was started on the fourth day after the operation, as jointly decided by the cardiology and neurosurgery team. As a bridge therapy, 2 × 0.6 cc subcutaneous enoxaparin was started at the end of the seventh day. Because of the planned Cesarean delivery, the 2 × 0.6 cc subcutaneous enoxaparin was continued within the therapeutic range, and checking of the anti-factor Xa level every 2 weeks was considered.

In conclusion, although the thrombogenic risk is high, it is vital to complete the reversal of warfarin anticoagulation in pregnant women with major bleeding, and practitioners should be aware of warfarin-associated intracranial hemorrhage management during pregnancy. Even though the mother should be the primary concern of the treatment approach, the foetal situation might also be kept in mind through a multidisciplinary approach.

## Data Availability

The datasets used and/or analyzed during the current study are available from the corresponding author on reasonable request.

## Abbreviations

MVT: Mechanical valve thrombosis
ICH: Intracranial haemorrhage
FFP: Fresh frozen plasma
LMWH: Low molecular weight heparin
UFH: Unfractionated heparin
